# Medical Jurisprudence

**Published:** 1850-11

**Authors:** 


*Medical Jurisprudence.* By ALFRED S. TAYLOR, F. R. S., Licentiate of the Royal College of Physicians, etc., etc., etc. Second American from the third London edition. With notes and additions by R. EGLESFELD GRIFFITH, M. D., etc. Philadelphia: Lea & Blanchard. 1850. 8 vo., pp. 670.

The first English edition of this valuable work appeared in 1844, and in the interim two large editions have been sold. The present is the second American, printed from the third English edition, the latter having been in a great measure re-written and much valuable matter added. The American Editor, the late lamented Griffith, a short time before his death added numerous notes and observations. The work thus purports to embrace all "that is important or useful in Legal Medicine, and all facts in relation to it, to the close of 1849." For sale by Phinney & Co.

